# Effects of Process Parameters on Structure and Properties of Melt-Blown Poly(Lactic Acid) Nonwovens for Skin Regeneration

**DOI:** 10.3390/jfb12010016

**Published:** 2021-02-26

**Authors:** Ewa Dzierzkowska, Anna Scisłowska-Czarnecka, Marcin Kudzin, Maciej Boguń, Piotr Szatkowski, Marcin Gajek, Kamil Kornaus, Magdalena Chadzinska, Ewa Stodolak-Zych

**Affiliations:** 1 Department of Biomaterials and Composites, AGH University of Science and Technology, 30-059 Kraków, Poland; pszatko@agh.edu.pl; 2 Department of Cosmetology, Academy of Physical Education, 31-571 Kraków, Poland; anna.scislowska@awf.krakow.pl; 3 Lukasiewicz Research Network-The Textile Research Institute, 92-103 Lodz, Poland; kudzin@iw.lodz.pl (M.K.); mbogun@iw.lodz.pl (M.B.); 4 Department of Ceramics and Refractories, AGH University of Science and Technology, 30-059 Kraków, Poland; mgajek@agh.edu.pl (M.G.); kornaus@agh.edu.pl (K.K.); 5 Faculty of Biology, Institute of Zoology and Biomedical Research, Jagiellonian University, 30-387 Kraków, Poland; magdalena.chadzinska@uj.edu.pl

**Keywords:** melt-blown nonwoven, Poly(lactide) acid, nonwoven tissue engineering scaffolds, skin tissue engineering, wound healing

## Abstract

Skin regeneration requires a three-dimensional (3D) scaffold for cell adhesion, growth and proliferation. A type of the scaffold offering a 3D structure is a nonwoven material produced via a melt-blown technique. Process parameters of this technique can be adapted to improve the cellular response. Polylactic acid (PLA) was used to produce a nonwoven scaffold by a melt-blown technique. The key process parameters, i.e., the head and air temperature, were changed in the range from 180–270 °C to obtain eight different materials (MB1–MB8). The relationships between the process parameters, morphology, porosity, thermal properties and the cellular response were explored in this study. The mean fiber diameters ranged from 3 to 120 µm. The average material roughness values were between 47 and 160 µm, whereas the pore diameters ranged from 5 to 400 µm. The calorimetry thermograms revealed a correlation between the temperature parameters and crystallization. The response of keratinocytes and macrophages exhibited a higher cell viability on thicker fibers. The cell-scaffold interaction was observed via SEM after 7 days. This result proved that the features of melt-blown nonwoven scaffolds depended on the processing parameters, such as head temperature and air temperature. Thanks to examinations, the most suitable scaffolds for skin tissue regeneration were selected.

## 1. Introduction

Polymer fibrous scaffolds can be obtained through a variety of techniques: melt-blown technology, electrospinning, phase separation, self-assembly or template synthesis [1]. For years researchers have been focusing on fabricating nonwoven tissue scaffolds via electrospinning (ES) [2,3]. However, electrospinning has weaknesses that make this method difficult to apply at the industrial scale as many polymers require the use of environmentally harmful organic solvents in order to obtain electrospinning solutions [4]. Moreover, the production of electrospun scaffolds is time-consuming and involves using high voltage. Additionally, a typical electrospinning process leads to the formation of dense fibers deposition [5]. With regard to the cell response, such a dense fibrous structure limits the cell infiltration, and thus only the suboptimal cell response is obtained. Therefore, nowadays researchers are focusing on developing a more economical and biocompatible method to produce scaffolds for skin tissue.

**Figure 1:**
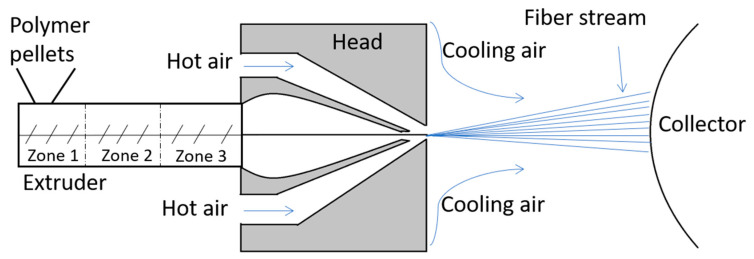
Meltblowing process diagram with the extruder and its zones: zone 1—the feed zone; zone 2—the transition zone; and zone 3—the metering.

Meltblowing is a one-step, solvent-free and high-throughput fabrication process that converts a solid polymer directly into a nonwoven mat (Figure 1). The polymer material is melted in the extruder in which polymer pellets are mechanically sheared with a rotation endless screw at a high temperature. The extruder has three different zones—the feed zone (1), the transition zone (2) and the metering zone (3). The molten polymer passes through a die with a number of small-diameter holes which turns the material into filaments [6]. Then the filaments are stretched by forced hot air onto a collector at high speeds, due to the vacuum suction system. The set-up size may vary from a bench top model to industrial production standards with fiber formation speeds up to 5000 m/min [7]. The process does not require devices operating at high voltage or solvents that might remain in the material. Similarly to electrospinning, the meltblowing parameters, such as the air flow, die-to-collector distance and die geometry, can be tailored to obtain desired physical characteristics. Melt-blown fibers are characterised by average diameters exceeding 5 µm and the extremely high ratio of surface area to volume [8].

One of the most commonly used polymers in the melt-blown technique is polypropylene (PP) [8,9] endowed with great rheological and physical properties. However, PP is produced from non-renewable resources and is more widely used in bone tissue engineering and suture than as a skin substitute [10]. Therefore, in skin tissue engineering, polylactic acid (PLA) could replace PP as it is biodegradable and more environmentally friendly. PLA is synthesized via the direct polycondensation of lactide acid or by the lactide ring-opening polymerization [11]. This aliphatic semi-crystalline polyester is easily processable and has good thermal plasticity [12]. Its glass transition temperature is 55–59 °C and has a melting point in the range of 174–184 °C. Such parameters allow successful PLA processing via the melt-blown method. Moreover, PLA is approved by FDA as a non-toxic material regarding both the human body and the environment [13].

Additional to the benefits of polylactide cited here, which allow for its use in medicine and implantology, PLA also has its limitations. These include biological inertness, dependent on the presence of enantiomers and molecular weight-degradation rate and, if the degradation rate is too high, degradation by products which strongly acidify the surroundings [14]. In extreme conditions, this can cause inflammation and necrosis of the surrounding cells [15]. However, due to the ease of processing PLA-based biomaterials by extrusion, injection molding, film casting, foaming, fiber spinning, electrospinning/melt electrospinning, and micro- and nano-fabrication techniques into various shapes and sizes, they have played a critical role in expanding the applications of these materials in biomedical application [16,17]. An attractive form of the material-fibrous scaffold with multidirectional arrangement of fibers, such as we get in the melt-blown technique, guarantees high porosity of about 90% and different size distribution allows us to obtain a high surface area in the scaffold. Such material parameters facilitate migration and penetration in the material by calls and water, which affects the kinetics of biodegradation (enzymatic/hydrolytic).

Therapeutic biomaterials facilitate wound healing processes. They can also support synthetic skin grafting and thus replace autogenous or allogeneic grafts [18]. The fibrillar structure and nanoscale architecture of the natural extracellular matrix (ECM) justifies the concept of applying fibrous substrates for skin regeneration [13,19]. Collagen and elastin are the two most important dermis ingredients that ensure its tensile and elastic properties [20]. In natural skin, the type I collagen fibers measure about 50–500 nm in diameter, the collagen type III-30–130 nm and the elastin fibers-between 100 and 200 nm. In laboratories, fibers of such diameters might be obtained via electrospinning. Yet despite the proper nanometric architecture, the substrates might lack adequate mechanical properties sufficient enough for skin regeneration [21]. Therefore, in order to obtain adequate mechanical properties, it seems reasonable to develop a combination of nanometric electrospun fibers and submicron or micrometric melt-blown (MB) fibers that will mimic the ECM structure. The combination of microfibers and nanofibers also provides the better cell infiltration and adhesion than either material itself [22]. Large open pores of the MB material enhances the cell infiltration, thus the nanofibrous architecture of the ES scaffold facilitates the cell adhesion and growth. The ES fibers are easily modified with substances accelerating wound healing [23], which is crucial to induce the desired cell behaviour, i.e., response, adhesion and migration on a given scaffold. Overall, the cell behaviour is conditioned by their sensitivity, size, matrix adhesion or filopodia formation [24]. For instance, the minimum fiber diameter for proper fibroblasts and keratinocytes adhesion and migration was proved to be 10–50 µm. The gaps (pores) between the fibers in reference to the pore size is also important, e.g., the range of 0–200 µm is suitable for fibroblasts to spin gaps between two fibers, while for keratinocytes it is 0–80 µm.

In this study, we aimed to compare eight PLA melt-blown nonwovens produced under different process conditions in order to identify the material best tailored for wound healing. We investigated how the temperature of the 3-zone extruder, the head and air affected the microstructural, thermal and biological properties of the melt-blown nonwovens. The microstructure was evaluated using the scanning electron microscope and the fiber diameters were established via micrographs analyses. The surface roughness was assessed with a laser microscope and the packing density was determined using the mercury porosimeter and gravimetric method. The phase transition temperatures were investigated by differential scanning calorimetry (DSC). The materials biocompatibility and the material–cell interactions were evaluated thanks to the keratinocytes and macrophage viability assessment.

## 2. Materials and Methods

Polylactide (PLA) Inego™ biopolymer 3251D, purchased from NatureWorks^®^ (Minnetonka, MN, USA), was selected for the scaffold investigations. Prior to the melt-blown processing, the PLA pellets were dried at 40 °C for 12 h to remove moisture.

The PLA nonwovens were produced via the melt-blown technique using a single-screw laboratory extruder (Axon, Limmared, Sweden) with a head with 30 holes, 0.25 mm in diameter each, a compressed air heater and a rotating drum (collector). PLA was fed to the extruder, where it was melted under the influence of the applied thermal energy and fed to the spinning head through the extruder. The high pressure polymer melt was “blown” through the dies. The nonwoven samples were deposited on the rotating drum. A summary of the processing parameters is presented in Table 1. The first stage of work on PLA nonwovens was to evaluate their homogeneity (comparable fiber sizes tested in several areas of nonwovens). The nonwovens that were tested were 28 cm × 35 cm in size. Selected areas (minimum 10 sites) of each nonwoven were evaluated and the mean diameter of at least 100 fibers were compared.

The microstructure properties of the nonwoven scaffolds were investigated by Scanning Electron Microscopy (SEM). The samples were coated with 10 nm gold layer using the rotary pump sputter coater (Leica EM ACE600, Wetzaler, Germany). The SEM observations were conducted with NOVA NANO SEM 200 (FEI, Hillsboro, OR, USA). At least 100 measurements were taken for each material. In order to measure the fiber diameter, the SEM images were analysed under the Fiji Lifeline software (ver. 22 December 2015). Based on these data, the average fiber diameters with standard deviation were calculated.

The basic weight of the manufactured samples was determined. The samples were cut into 100 mm × 100 mm squares and weighed. Based on the obtained results, the surface weight of the product was calculated according to Equation (1).

(1) $$Basic\;weight=\frac{mass\;of\;the\;sample\;\left(g\right)}{sample\;area\;\left(m^{2}\right)}$$

The porosity of the nonwoven scaffolds was estimated using the gravimetric method (2) [25].

(2) $$Porosity\;\left(\%\right)=\left(1-\left(\frac{Apparent\;density\;of\;scaffold\;\left(\frac{g}{cm^{3}}\right)}{Average\;bulk\;density\;of\;the\;polymers\left(\frac{g}{cm^{3}}\right)}\right)\right)\times 100$$

The apparent density was calculated based on Equation (3).

(3) $$Apparent\;density\;of\;scaffold=\frac{mass\;of\;the\;sample\;\left(g\right)}{sample\;thickness\;\left(cm\right)\times sample\;area\left(cm^{2}\right)}$$

The scaffold mass was evaluated using scales (QUINTIX 125D 1CEU, Sartorius, Goettingen, Germany; precision of 0.00001 g). The membrane thickness was measured by the digital microscope VHX-6000, Keyence, Japan. The average bulk density of PLA was 1.24 g/cm^3^.

The diameter and distribution of open pores in the final materials was determined with the mercury porosimeter PoreMaster 33 (Quantachrome Instruments, Ashland, VA, USA). The sensitivity range of the sizes measured by the pore device was 7 nm–1 mm. The average weight of the tested samples was 150 mg.

The thermal properties were investigated by means of the differential scanning calorimetry (DSC1, Mettler Toledo, Columbus, OH, USA). The heating rate was 10° min^−1^ with dynamic nitrogen atmosphere for the sample mass of 5 mg placed in aluminum pans.

The scaffold roughness was examined via the laser microscopy (Olympus OLS4000, Osaka, Japan). Each sample was coated with the 10 nm gold layer using the rotary pump sputter coater (Leica EM ACE600, Wetzaler, Germany). The examined area was 200 µm × 200 µm and ten measurements were taken at different places of the sample to obtain average roughness (R_a_). R_a_ parameters were calculated and at least 10 measurements were taken for each material with standard deviation.

Biological studies were carried out using human keratinocytes HaCaT (cell line from Primary Epidermal Keratinocytes from ATCC^®^ PCS-200-011™, Manassas, VR, USA) and murine RAW 264.7 macrophage-like cells (from ATCC^®^ TIB-71™, Manassas, VR, USA). The 8 discs of each sample with a diameter of 16 mm were subjected to cellular analysis, both 8 for viability and cytotoxicity, each day. The cells were cultured in 75 mL plastic bottles (Nest Scientific Biotechnology, Wuxi, China) in the DMEM culture medium enriched with glucose, L-Glutamine (Lonza, Basel, Switzerland), 10% fetal bovine serum (Biowest, Nuaillé, France) and the 5% antibiotic solution containing penicillin (10 UI/mL) and streptomycin (10 mg/mL, both Lonza, Switzerland). The cells were cultured in the incubator (Nuaire, Plymouth, MN, USA) at 37 °C and 5% of CO_2_. Every 2–3 days, when the cells formed high confluence monolayers, the keratinocytes culture was passaged by trypsinization (0.25% solution of trypsin; Sigma-Aldrich, Hamburg, Germany) and the macrophages culture by scraping.

For the biological studies the polymeric scaffolds were washed in 70% ethanol, then sterilized with UV irradiation (20 min for each side) and placed at the bottom of 24-well dishes (Nest Scientific Biotechnology, Wuxi, Jiangsu, China). The cells were harvested after 7 to 10 passages, counted in the Bürker’s hemocytometer, diluted to 1 × 10^4^ cell/mL and then placed in the 24-well culture dishes (Nest Scientific Biotechnology, Wuxi, Jiangsu, China) containing discs of the tested biomaterials. The nest tissue culture polystyrene (Nest Scientific Biotechnology, Wuxi, Jiangsu, China) was used as the control material (CTR). In such conditions the cells were cultured for 3 or 7 days. Subsequently, the morphology of cells adhering to the polymeric scaffold was observed under the inverted microscope (Jenamed SH50, Jena, Germany).

The viability of RAW 264.7 and HaCaT were determined with the Vialight^®^Plus test (BioAssay Kit, Lonza, Switzerland). The cytotoxicity of the materials was determined using the ToxiLight^®^ test (BioAssay Kit, Lonza, Switzerland). The intensity of light emitted by the cells containing the added color factor was measured with a FLUOStar Omega luminometer (BMG Labtech, Ortenberg, Germany). All the aforementioned tests were performed according to the manufacturers’ protocols.

The cell morphology after the 7th day was evaluated by SEM. The nonwoven scaffolds were fixed in 2.5% glutaraldehyde (Sigma Aldrich, London, UK) for 2 h. Next, the samples were dehydrated in the series of the ethanol solutions (50, 70, 96%, Avantor, Poland) 3 times for 3 min each and left to dry. Finally, the samples were mounted on the SEM holder with a carbon tape and coated with the gold layer of the same parameters as for the fiber microstructure observation.

The one-way ANOVA was used to determine the significance level for biocompatibility tests of the fibrous membranes. The statistical significance was evaluated at *p* < 0.02. The variance analysis was performed at Origin 2020.

## 3. Results

### 3.1. Nonwoven Scaffold Morphology

**Figure 2:**
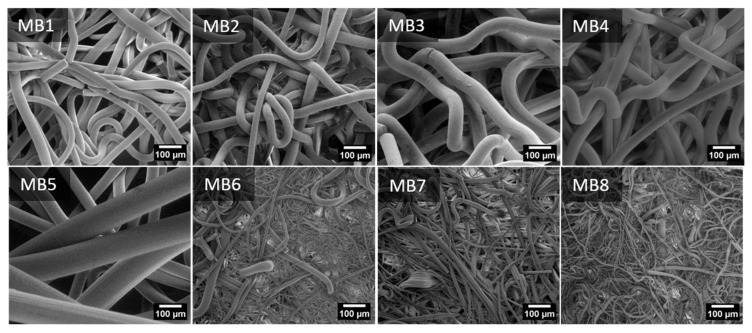
Scanning electron microscopy photography of nonwoven scaffold MB1-8.

**Figure 3:**
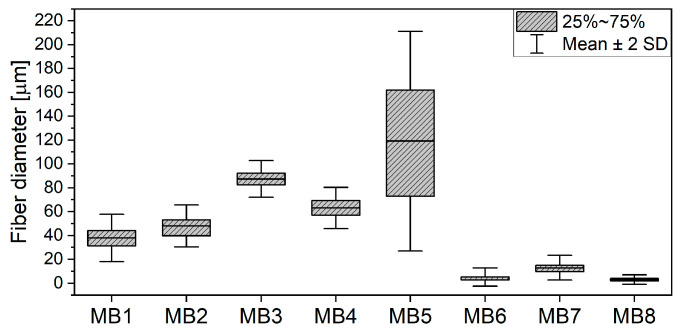
Fiber diameter distribution of PLA melt-blown scaffolds.

The SEM micrographs of the PLA MB nonwoven are shown in Figure 2. For all the materials, the fibers orientation was random and the fibers were strongly tangled. The MB1-MB5 fibers had a smooth surface, yet elliptical inequalities were observed for the MB5 sample. Pores–the areas between fibers–were seen on the MB6 surface. Numerous fractured fibers were observed for the materials of the smaller fiber diameters (MB6–MB8). The fiber diameter distributions (Figure 3) are presented as box plots with an average fiber diameter, the interquartile interval and standard deviation. There were three groups of the tested fibers distinguished after the analysis of diameter average values. In the first group there were fibers with an interquartile interval about 20 µm and the average fiber diameters of 40–90 µm. In the second group there was the MB5 sample with the widest interquartile range of 80 µm and the average fiber diameter of 119 µm. The narrowest interquartile interval was characteristic for the third group (MB6–MB8) where the average fiber diameters ranged from 3 to 12 µm.

**Figure 4:**
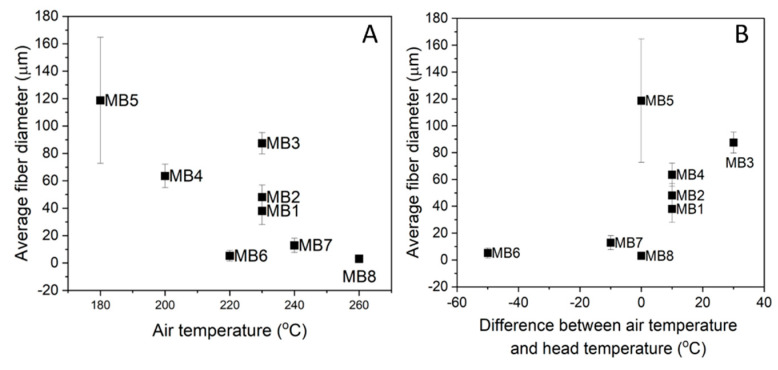
Fibers diameter versus: (**A**) air temperature; and (**B**) difference between air temperature and head temperature for PLA melt-blown scaffolds.

Figure 4A presents the average fiber diameter with regard to the air temperature process parameter. It can be noticed that the lowest air temperature generated the largest fibers (MB5–119 µm) and the highest air temperature, the smallest ones (MB8–3 µm). The fiber size distribution of the other materials depended on the gradient between the head (nozzle) temperature and the air temperature at which the fibers were solidified (Figure 4B). The small-diameter fibers up to 15 µm (MB6, MB7) were formed when the air temperature was lower than the head one. It is worth noting that this temperature was still higher than or equal to 220 °C. The air temperature higher than the head temperature generated the larger-diameter fibers (MB1–MB4). In the case of MB5 and MB8 there was no difference in the air and head temperatures.

**Figure 5:**
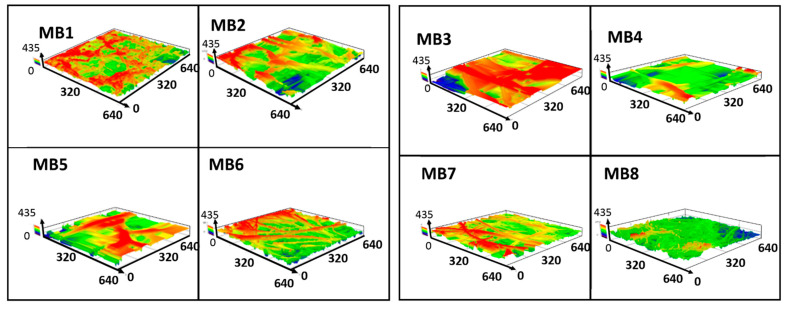
PLA nonwovens surface profile of the rough surface found in 3D laser microscopy.

The surface roughness of the melt-blown materials was evaluated by means of the average roughness (Ra). This parameter of each scaffold is listed in Table 2. The samples revealed significant differences in roughness, with the greatest difference reaching 100 µm. The roughness above 100 µm was observed for the fibers with the average diameter above 60 µm and the highest Ra of 158 µm-for MB3. The fibers below 15 µm in diameter showed the lowest roughness values at around 50 µm. The MB5 material with the largest fiber diameter did not show the highest roughness due to the wide size distribution. The complex 3D architecture of nonwoven scaffolds was confirmed in the topographic maps, highlighting the membrane surface (Figure 5).

Designing scaffolds for tissue engineering requires establishing the material porosity and the pore size. As the evaluation of the fiber size distribution and surface roughness did not provide the data regarding the material porosity, the materials in our study were also examined by means of the mercury porosimetry.

### 3.2. Porosity and Pore Size Distrubution

The values of apparent density, basic weight and porosity of the melt-blown scaffolds are presented in Table 3. The basic weight as a parameter of the fiber packing density on the sample surface showed significant variations between the tested materials. The lowest fiber packing was observed for MB1 and MB6 and the highest for MB2. On the basis of the other results, two groups were distinguished: the MB1–MB6 samples with porosity below 95% and apparent density of scaffold above 6 g/m^3^ and MB6–MB8 with a higher porosity and a lower density. The highest apparent density, and thus the lowest porosity, was determined for MB3, the material with the highest roughness and one with the highest average fiber diameters. The MB6–MB8 samples showed lower density values and higher porosity. It might be concluded that these materials have a well-developed structure with inter-fiber connections, taking into account the smaller fiber diameters, the sample mass similar to the other materials and the lower thickness value.

**Figure 6:**
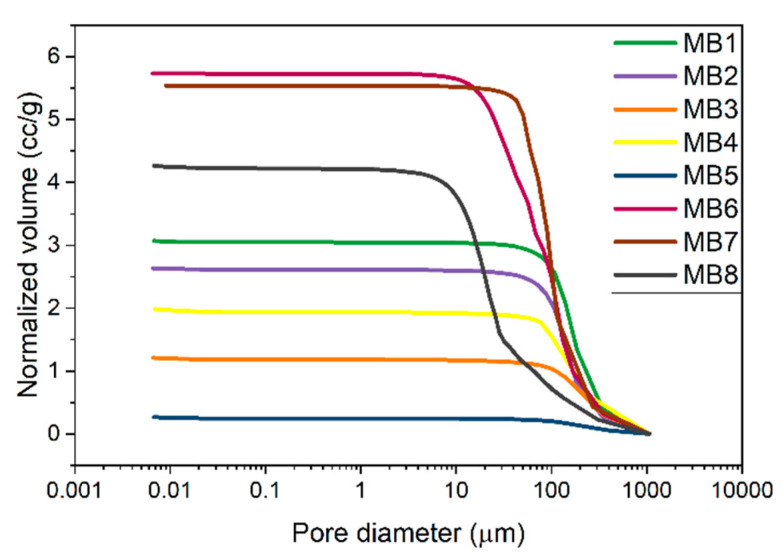
Cumulative curve of the pore size distribution of melt-blown nonwovens.

**Figure 7:**
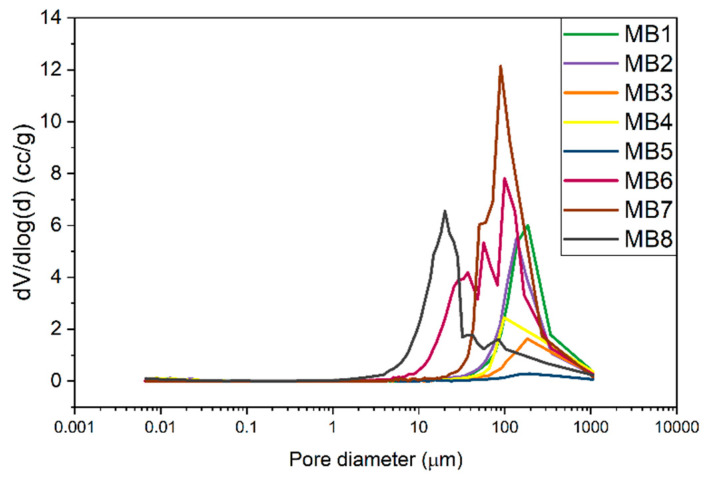
Frequency curve of the pore size distribution of nonwovens.

The cumulative curve of the pore size distribution showed the dependence of the pore volume on the pore diameter. Figure 6 gives information about the open pore volume per 1 g of the sample. The values in the diagram for all the materials coincide with the porosity from Table 3. The MB6, MB7, and MB8 samples showed the highest pore volume per 1 g of material (e.g., open porosity) while the smallest values were observed for MB3 and MB5. Figure 7 presents the 1st derivative of the cumulative curve in Figure 6, describing the frequency as a function of the pore diameter. This curve enabled us to determine the volume of pores of a specific diameter as well as the modal pore diameter.

The pore size range for the MB1–MB5 and MB7 materials was similar and spanned from 40 to 400 µm. MB8 exhibited the smallest pores of 5–30 µm and fewer pores of 30–300 µm. The wide pore size distribution was observed for MB6, ranging 10–300 µm.

### 3.3. Thermal Analysis

**Figure 8:**
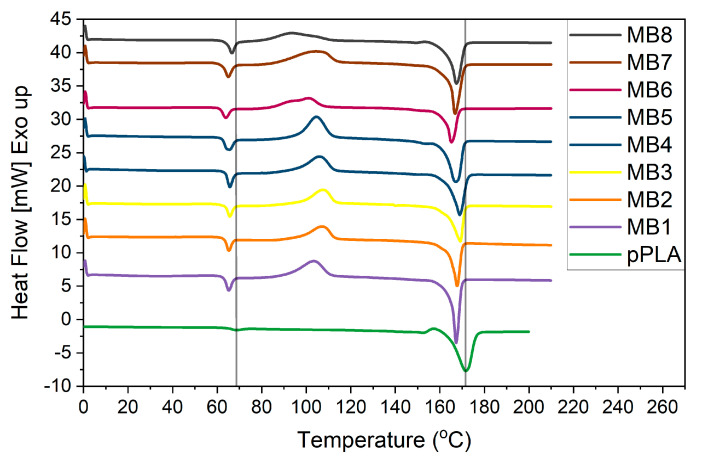
DSC heating scans of melt-blown nonwovens.

The DSC results for the melt-blown PLA scaffolds and PLA pellet (pPLA) are presented in Figure 8. The pPLA thermograph exhibited the glass transition temperature (T_g_) at 64 °C and a melting temperature (T_m_) at 164 °C. On the other hand, all the melt-blown PLA nonwoven exhibited a shift in T_g_ and T_m_ values towards lower temperatures. Besides the shifted temperature of T_g_ and T_m_, there were two other distinct features observed: the exothermic peak associated with cold crystallization around 90 °C and another exothermic peak near 155 °C. The major difference between the pPLA and all the MB scaffolds was the lack of an exothermal peak around 90 °C for pPLA. The MB materials also revealed a lower second exothermal peak. Within the MB scaffold group, MB6, MB7 and MB8 displayed the lower T_c_ temperature with the broadened and asymmetric peaks. The pPLA material displayed the melting process over a wider temperature range of the melting process. All the specific temperature values are summarised in Table 4. Moreover, the crystallinity degree (X_c_) was determined on the basis of the melting enthalpy of fully crystalline polylactide, which is 93 J/g [26].

### 3.4. Cellular Analysis

**Figure 9:**
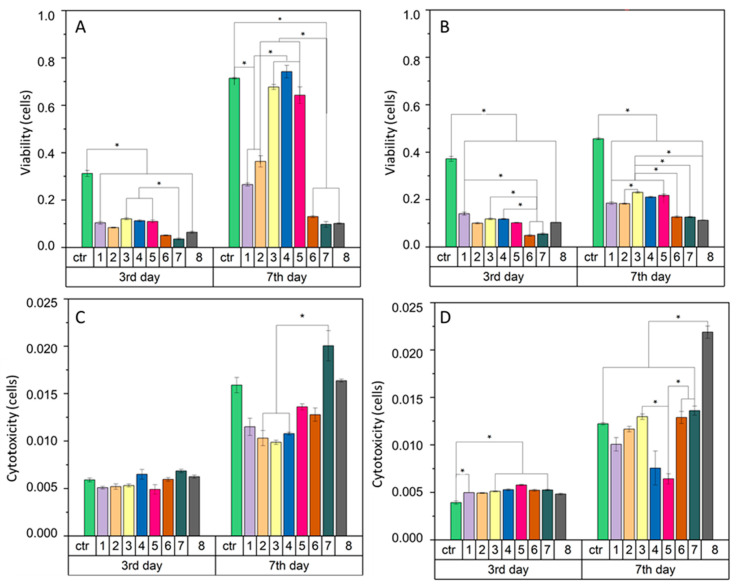
Cell viability across different nonwoven scaffolds of: (**A**)—HaCaT human keratinocytes cells, (**B**)—RAW 264.7 murine macrophage-like cells and cytotoxicity (**C**,**D**) for the same cells, respectively. The unit of cells is appropriately converted from the luminescence on 3rd and 7th day relative to the day the culture started. Standard error of the mean is represented as error bars. * statistical difference between scaffold types connected by bars (*p* < 0.02).

In general, the interaction of fibrous membranes with cells is determined by means of the cell viability assessment. A high viability rate in relation to the total number of cells indicates a good cellular response. In our study the keratinocytes viability (Figure 9A) on the 3rd day of culture was very low for individual samples. The results were more than two times lower than the control values. The cell activity and viability increased on the 7th day of the keratinocytes culture. The MB3, MB4, and MB5 materials showed the high cell viability rate, at a level similar to the control. The viability increase was also observed for MB1 and MB2, while for MB6, MB7 and MB8 the cell viability index slightly changed. The analysis of the macrophages viability (Figure 9B) indicated a low rate. On the 3rd day of culture the cell viability index for each material was two times lower than the control value. On the 7th day, the macrophage viability on the MB samples increased and these results were significantly different to the control.

Cellular cytotoxicity (Figure 9C,D) was investigated to determine the detrimental effect of the material on cells. The melt-blown fibers showed no significant differences in cytotoxicity with both keratinocytes (Figure 9C) and macrophages (Figure 9D) on the 3rd day of the cell culture. Changes in cytotoxicity were visible only on the 7th day for both cell types. The MB7 and MB8 materials revealed the highest cytotoxicity for keratinocytes, higher than or equal to the control, respectively. For the remaining materials, the cytotoxicity index was lower than for the control. However, for the macrophages culture, on the 7th day the cytotoxicity value of MB8 exceeded the values of the other samples and MB4 and MB5 showed the lowest ones.

**Figure 10:**
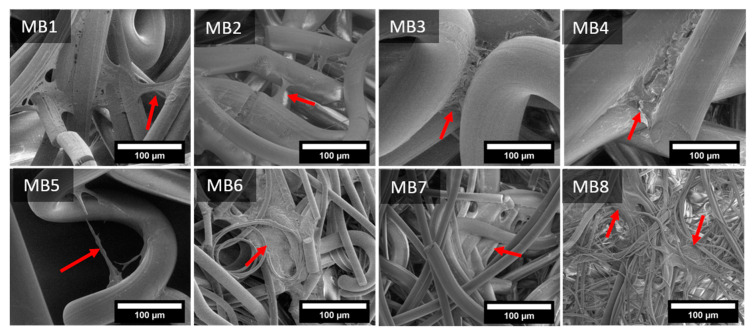
SEM microphotographs of keratinocytes spread on melt-blown nonwovens on 7th day of culture. The arrow indicates keratinocytes flattened on fibers or migrating to adjacent fibers.

**Figure 11:**
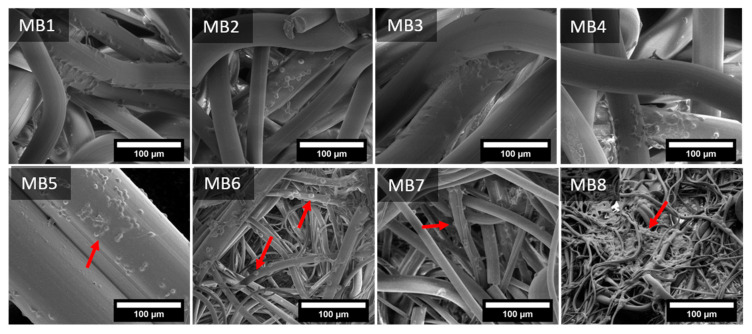
SEM microphotographs of macrophages spread on melt-blown nonwovens on 7th day of culture. The arrow indicates macrophages flattened out on a fiber surface.

The cells interactions with the fibrous scaffolds was examined by SEM. Figure 10 and Figure 11 present the images of keratinocytes and macrophages spread out on all the materials after 7 days of incubation.

Keratinocytes spread at the junction of two or more fibers, where they began to flatten out and migrate to adjacent fibers, depending on the particular samples. Cellular migration on MB1–MB5 seemed to occur more often in the transverse direction than along the fiber. For MB6–MB8 the cells spread both laterally and along the fibers. Moreover, the fiber diameters and the space between them was small enough for the cells to evenly cover also many adjacent fibers.

Macrophages adhered evenly over the entire surface of the samples, covering them on each side. Dozens of single cells were observed on the surface of larger-diameter fibers (MB1–MB5), while on the smaller-diameter fibers (MB6–MB8), the macrophage colonies were visible both on the fibers and between them. It seems that macrophages recognized the larger-diameter fibers as a flat surface which they colonized, creating connections between the fibers. On the other hand, the small-diameter fibers (MB8) facilitated the attachment of cells to entangled fibers, supporting the cell proliferation.

## 4. Discussion

Polymeric fibrous materials are known as excellent scaffolds in tissue engineering. In the current study we produced nonwoven fibrous scaffolds using a melt-blown technique. The temperature differences in particular zones of the extruder generated various fluidity values of the polymer melt and their different susceptibility to fiber formation. The viscosity of the polymer melt affected the polymer stream behavior both in the head die and in the space between the spinning head and the collector. Apart from applying different temperatures in the 3-step extruder, the temperature of the head and air were also adapted. As a result, we obtained eight materials characterized by different morphologies, topographies and thermal properties.

Taking into account all the process parameters, the die temperature had the greatest influence on the fiber diameter. The finest fibers were achieved at the die temperature above 260 °C (MB6–MB8) as fibers elongated more easily near to the die and the polymer viscosity decreased at higher temperatures [27]. The test results also suggested that the entire die-collector space was influenced by the die temperature. However, the thermal process characteristics of each polymer melt and further changes in the air temperature also affected the fiber size. For instance, MB6 had larger fibers than MB8 despite the higher die temperature. The temperature of the last stage of extruder for MB8 was 55 °C higher than MB6 (Table 1). The air temperature in the case of MB8 did not change as drastically as for MB6 (Figure 4B). The same correlation was retained for the scaffolds with thicker fibers, except for MB3, whose increased melt flow rate significantly affected the fiber diameter.

The process parameters were selected to obtain the round cross-section fibers since this shape is more favorable to the cells attachment and proliferation in comparison to the snowflake-shaped fiber [28]. The head temperature below 200 °C (MB4, MB5) resulted in the uneven structure in fibers surface. The not high enough temperature resulted in partial clogging of the polymer in the head die [29]. The last stage in the fiber heating is hot air and as the fibers travel to the collector, they are cooled by the much lower room temperature. It was confirmed that during this passage fine fibers are not as hot as coarse fibers [27]. Consequently, during the passage, coarse fibers are more likely to fuse than fine fibers. Fiber fusion results in broader fiber diameter distributions, which was observed for MB1-MB5 scaffolds. Additionally, these materials revealed the higher average roughness (Ra) in comparison to thinner fibers (Table 2) and the fiber diameter and fiber orientation are responsible for the porosity of the entire material.

A porous material should be a framework with connected networks suitable for the local cell growth and tissue organization that permits the cell attachment, distribution and proliferation [30]. The pore size affects the cell–cell reaction in the initial phase of the cultivation, which consists of adhering and cell migration over the surface of the scaffold. Moreover, the pore size is also responsible for providing cells with enough space to grow inside the material. Generally, different tissues require different pore sizes. The skin tissue needs the pore size below 160 µm, which is suitable for fibroblasts and the connective tissue is formed with the pores below 100 µm. However, the vascular infiltration which provides nutrients to the new tissue is only possible at 10,000 µm. The porosity of the melt-blown nonwovens was over 90%. The literature confirms that the porosity of scaffolds required for different cellular activities is in the 70–99% range [31] and the cells development and tissue rebuilding are strongly dependent on pore sizes [31]. Most of the materials tested in this study revealed the pore size around 100 µm, which may be sufficient for skin rebuilding. The pore sizes for the materials with the highest cell viability (MB3–MB5) were in the 40–400 µm range. In addition, keratinocytes were able to span the gaps between two fibers up to 160 µm, as shown in the SEM micrography (Figure 10-MB5). This was twice the value of the distance from the previously performed tests [24].

The melt-blown technique imposes many changes on the material structure. In the first phase, the polymer pellets are heated with the screw above the glass transition temperature (Tg). At this point, the polymer chains are free to move and ready to crystallize. The next stage is the polymer melt passage through the hot die where it is again subjected to the force delaying the cold crystallization. The hot air stream blowing the polymer melt at high speed, causing the flow shear. The hot fibers coming out of the die are rapidly cooled down to the room temperature of the collector. This process is non-isothermal crystallization and both the shear flow and the high initial temperature of the polymer melt delays the cold crystallization [32]. In the case of the polylactide, the long half-life of this polymer crystallization also plays an important role [33,34]. The absence of the peak around 100 °C proved that there was no α-form of crystalline morphologies in pPLA. However, the presence of an exothermal peak at 157 °C is attributed to the β-form, which is less ordered and transformed into an α-form [32]. Yet, processing PLA to obtain melt-blown fibers greatly enhanced the non-isothermal crystallization rate. Table 4 shows that the Tc value of the PLA fiber was between 81 to 97 °C and it was correlated with the fiber diameter. As previously described, three subgroups were distinguished based on the fiber diameter. The first group was the MB1–MB4 scaffolds with the similar and equally symmetrical thermal range of exothermic peaks. In the second group, there was MB5 with the most symmetrical exothermic peak. The third group with the wider and asymmetric cold crystallization peak was assigned to the MB6–MB8 scaffolds. It has been shown that there are areas within the fibers which can be distinguished by their crystallinity (higher in the centre area and lower in the edge of fibers) [35]. This effect resulted from the changes in the chain microenvironments inside the fibers, which depend on the shear flow of the manufacturing process [32,35]. The size of the inner and edge regions of the fiber, and hence the fiber diameter, influences the cold crystallization extent. Therefore, the difference in the ranges for cold crystallization depends on the fiber diameter. In our study this was confirmed by the increase in the onset of cold crystallization in the case of the larger-diameter fibers (MB1–MB5) (Figure 8).

Crystallinity is one of the material variables that affect physical-mechanical and biodegradability properties [27,36,37]. High crystallinity of PLA fibers implies high stiffness and low ductility of the material [38]. Apart from affecting the material physical properties, crystallinity has an influence on the cell attachment, growth and proliferation [39,40,41]. The complex nanostructure of polymer crystallites influences the cellular response due to the proper material topography and the crystals size. Cells recognize the structure which has dimensions similar to their size (10–100 µm) [39]. Therefore, it is worth considering the crystallite size in terms of the cellular response. It was shown that each type of cell reacts differently to the material crystallinity. The thermal forming of fibers leads to changes in their structure. A more crystalline phase should be formed inside the fiber (the core has more difficult cooling than the surface of fibers). The outer layer of the fiber, which is cooled faster, should maintain a more amorphous (disorderly) character as is the case with 3D printing films. In the case of the meltblown molding technique, thermal degradation of PLA additionally occurs as a result of keeping the polymer at a temperature above 260 °C and the shearing effect of the screw. As a result, we get a polymer with a shortened chain, which facilitates the formation of spherules in the surface layer (MB6, MB8-among which the crystallinity of the polymer increases). Such behavior was analyzed by researchers with reference to thin PLA and PGLA films [39,40,41]. They indicated that the heterogeneity of the surface caused by thermal treatment affects the cellular response. Sensitive cells (e.g., hepatocytes) prefer to respond more quickly on the crystal surface, showing a stronger secretion (enzyme from cytochrome P-450) but they also quickly detach from it, which leads to their necrosis. Less sensitive cells (e.g., fibroblasts) spread more slowly on stronger crystalline surfaces, but after reaching their correct morphology, they show a higher activity than on amorphous media [40,41]. In our study, keratinocytes were characterized by higher vitality and more active metabolism on more amorphous materials (of lower crystallinity). Macrophages, on the other hand, preferred fibers with a higher degree of crystallinity, but also showed higher cytotoxicity. The study was carried out in two time intervals, so no cell necrosis was observed, and fewer cells were visible on the surface of the fibers.

Biological research using cell lines is widely carried out in the field of electrospun fibers [42,43,44] as melt-blown scaffolds could be produced on a large scale and used as medical materials. In our research, the different behaviours of the two cells types significantly depended on the fiber size and the surface roughness. Keratinocytes, due to their small size, prefer nanometric fibers [45]. The fibers we investigated were of three magnitudes, but still the characteristic behavior of keratinocytes was noticed. The surface roughness of the largest-diameters fibers (MB3, MB4, MB5) was not an important factor since keratinocytes perceived these large fibers as a flat surface. Keratinocytes prefer flat surfaces because they create many points of adhesion, which increases their activity and proliferation. It could also be seen in the nonwovens with nanometric diameters [45]. The highest cytotoxicity was demonstrated by MB7 and MB8 characterized by higher crystallinity (more than 50%). As far as macrophages are concerned, these cells are essential initiators of wound healing. They are of great importance in the process of tissue regeneration, therefore it is extremely important to study their cellular response to scaffolds. Macrophages reveal two stages of polarization (from M1 to M2) [46]. Firstly, classically activated macrophages (M1) are responsible for production of pro-inflammatory cytokines (Il-1β, IL-6 and TNF-α). Secondly, polarized macrophages (M2) express their own cytokine patterns (CD163, CD206, IL-10) and they exhibit more pro-healing properties. The spontaneous polarization might occur depending on the biomaterial architecture [47]. Human macrophages with elongated phenotypes were proven to induce polarization to M2. The smaller pore size of 40 µm and the fiber diameter around 3 µm promoted the elongation and the M2-like polarization. In our study, after 7 days of cultivation on the nonwoven scaffolds, macrophages showed a round shape. The only material confirming the better cell elongation was MB8, characterized by the thinnest fibers around 3 µm (Figure 3) and the largest pore distribution of 6–40 µm (Figure 7). However, MB6 also revealed the highest cytotoxicity among all the tested materials, probably related to the crystallinity degree. Other materials with thicker fibers were not suitable for spreading macrophages on the three-dimensional fiber scaffold. Macrophages are limited to interaction with a single fiber only. In future research, it would be necessary to study cytokine markers.

## 5. Conclusions

In our study we successfully manufactured polylactic acid nonwovens under different processing conditions. We proved that the morphology, thermal properties and cellular response of the materials were dependent on the processing parameters, such as the temperatures of the 3-step extruder, head and air. Moreover, the fiber size, roughness and porosity was closely related to the process temperatures. It was proven that the porosity of the obtained materials may support the skin tissue regeneration process. The correlation between cell viability and pore size/fiber diameter cannot be answered clearly. On the one hand we see such a correlation: looking only at the cell viability index and the fiber diameter, there is a correlation between them—the larger the fiber, the greater the cell viability. However, this cannot be viewed in such a narrow sector. As we show in other part of the manuscript, interactions between cells and materials have a much more complex dimension. It has been suggested that the crystallinity of the scaffold also plays an extremely important role.

The best results of keratinocyte viability were demonstrated for MB3 and MB4 materials. These cells showed an elongated morphology on the single fiber and also joined the adjacent fibers. On the other hand, it is known that the cellular response is a complex process dependent on many material features. To describe the behavior of cells on the basis of only one parameter can be misleading. Only a comprehensive approach to material properties gives a complete information of its application possibilities. The starting point here is the processing that determines the final characteristics of the material. This starts with the identification of properties that are the result of processing. In order to improve the material properties, there is still a possibility to modify the surface chemically. The other approach might be to create a new material combining both nano and micro fibers endowed with more adhesion points and better cell elongations. The results we obtained so far are promising regarding the material applicability in tissue engineering. The production method we developed offers the possibility to transfer the research from the laboratory to commercial applications.

## Figures and Tables

**Table 1:** Meltblowing process conditions for PLA.

Processing Parameter	MB1	MB2	MB3	MB4	MB5	MB6	MB7	MB8
Extruder zone 1 (°C)	195	195	190	180	170	195	195	195
Extruder zone 2 (°C)	230	230	195	190	175	200	230	245
Extruder zone 3 (°C)	235	235	210	190	180	205	245	260
Head temperature (°C)	220	220	200	190	180	270	250	260
Air temperature (°C)	230	230	230	200	180	220	240	260
Air flow (m^3^/h)	7–8	7–8	7–8	7–8	7–8	7–8	7–8	7–8
Melt flow rate (g/min)	5	5	8	5	5	5	5	5
Hole diameter (mm)	0.25	0.25	0.25	0.25	0.25	0.25	0.25	0.25
Die to collector distance DCD (cm)	26	26	26	26	26	26	26	26

**Table 2:** Average roughness (Ra) profile of melt-blown scaffolds with standard deviation.

Roughness	MB1	MB2	MB3	MB4	MB5	MB6	MB7	MB8
Ra (µm)	70.32 ± 12.38	91.41 ± 17.92	158.01 ± 27.90	118.44 ± 31.10	101.69 ± 20.39	54.24 ± 11.23	47.95 ± 12.53	48.89 ± 7.29

**Table 3:** Total porosity, apparent density, basic weight of melt-blown nonwovens.

Material	Weight (g)	Thickness (cm)	Basic Weight (g/m^2^)	Apparent Density of Scaffold (g/cm^3^)	Total Porosity (%)
MB1	2.23 × 10^−2^	4.09× 10^−2^	129.7	6.79 × 10^−2^	94.52
MB2	7.04 × 10^−2^	1.24 × 10^−1^	335.6	7.07 × 10^−2^	94.30
MB3	3.85 × 10^−2^	6.32 × 10^−2^	195.5	7.58 × 10^−2^	93.89
MB4	3.84 × 10^−2^	6.68 × 10^−2^	195.1	7.15 × 10^−2^	94.23
MB5	2.08 × 10^−2^	4.08 × 10^−2^	242.3	6.34 × 10^−2^	94.88
MB6	2.32 × 10^−2^	1.79 × 10^−1^	130.9	1.61 × 10^−2^	98.70
MB7	3.53 × 10^−2^	2.36 × 10^−1^	162.0	1.86 × 10^−2^	98.50
MB8	2.90 × 10^−2^	2.00 × 10^−1^	146.5	1.80 × 10^−2^	98.55

**Table 4:** Summary of characteristic temperature values of nonwoven scaffolds measured by DSC.

Material	T_g_ (°C)	T_c_ (°C)	H_c_ (J/g_PLA_)	T_m_ (°C)	H_m_ (J/g_PLA_)	X_c_ (%)	C_p_ (J/gK)
MB1	62.81	91.54	32.54	164.75	44.89	48.27	0.46
MB2	63.16	96.84	29.85	164.10	44.52	47.87	0.59
MB3	63.52	97.61	32.35	162.90	40.12	43.01	0.41
MB4	63.58	95.89	26.61	163.61	40.20	43.23	0.44
MB5	62.12	96.59	28.52	161.77	44.94	47.31	0.40
MB6	61.21	83.58	28.29	162.14	39.21	42.16	0.36
MB7	62.15	86.81	33.08	164.07	48.58	52.24	0.43
MB8	63.97	81.07	27.30	163.44	47.94	51.55	0.58
pPLA	64.28	-	-	164.25	44.13	47.45	0.11

## Data Availability

The data presented in this study are available on request from the corresponding author.
